# Gender and age differences in components of traffic-related pedestrian death rates: exposure, risk of crash and fatality rate

**DOI:** 10.1186/s40621-016-0079-2

**Published:** 2016-06-10

**Authors:** María Ángeles Onieva-García, Virginia Martínez-Ruiz, Pablo Lardelli-Claret, José Juan Jiménez-Moleón, Carmen Amezcua-Prieto, Juan de Dios Luna-del-Castillo, Eladio Jiménez-Mejías

**Affiliations:** 1 Doctorate Program in Clinical Medicine and Public Health, University of Granada, Granada, Spain; 2 Department of Preventive Medicine and Public Health, School of Medicine, University of Granada, Avda. de la Investigación, 11, 18016 Granada, Spain; 3 Centros de Investigación Biomédica en Red de Epidemiología y Salud Pública (CIBERESP), Barcelona, Spain; 4 Department of Biostatistics, School of Medicine, University of Granada, Avenida de Madrid 11 18012 Granada, Spain

**Keywords:** Pedestrian, Age, Gender, Exposure, Crash, Fatality, Mortality

## Abstract

**Background:**

This ecological study aimed i) to quantify the association of age and gender with the three components of pedestrians’ death rates after a pedestrian-vehicle crash: exposure, risk of crash and fatality, and ii) to determine the contribution of each component to differences in death rates according to age and gender in Spain.

**Methods:**

We analyzed data for 220 665 pedestrians involved in road crashes recorded in the Spanish registry of road crashes with victims from 1993 to 2011, and a subset of 39 743 pedestrians involved in clean collisions (in which the pedestrian did not commit an infraction). Using decomposition and quasi-induced exposure methods, we obtained the proportion of increase in death rates for each age and gender group associated with exposure, risk of collision and fatality.

**Results:**

Death rates increased with age. The main contributor to this increase was fatality, although exposure also increased with age. In contrast, the risk of collision decreased with age. Males had higher death rates than females, especially in the 24–54 year old group. Higher fatality rates in males were the main determinant of this difference, which was also related with a higher risk of collision in males. However, exposure rates were higher in females.

**Conclusions:**

The magnitude and direction of the associations between age and gender and each of the three components of pedestrians’ death rates differed depending on the specific component explored. These differences need to be taken into account in order to prioritize preventive strategies intended to decrease mortality among pedestrians.

**Electronic supplementary material:**

The online version of this article (doi:10.1186/s40621-016-0079-2) contains supplementary material, which is available to authorized users.

## Background

Pedestrian deaths after a crash make a large contribution to total crash mortality rates in many countries, especially low-income ones (Global Health Observatory WHO 2010; WHO 2013). The association of male gender and older age with higher pedestrian crash death rates (PCDR: deaths of pedestrians involved in road crashes/total population) is well known (Chang 2008; Koepsell et al. 2002; Yannis et al. 2011). However, few studies have analyzed the separate contributions of age and gender to the three main determinants of PCDR: the amount of exposure to crashes, the risk of collision with a vehicle adjusted by exposure, and pedestrian fatality rate after collisions. A recent study by Zhu et al. (2013) used a decomposition method to determine the contribution of these three components to the excess PCDR observed for male pedestrians; these authors used the number of kilometers walked as a measure of pedestrians’ exposure. However, the amount of pedestrian exposure to collisions with a vehicle should be restricted to the time during which there is a real risk of collision with another vehicle, i.e., when pedestrians are crossing or walking along roadways (Keall 1995; Lassarre et al. 2007).

In the present study we combined the decomposition method (Dellinger et al. 2002; Li and Baker 1996; Zhu et al. 2013) and the quasi-induced exposure method (Lardelli-Claret et al. 2006; Lenguerrand et al. 2008) to quantify the association of age and gender with each of the three components of PCDR (exposure, risk of crash and fatality rate), as well as to determine the contribution of each of these components to differences in PCDR according to age and gender in Spain from 1993 to 2011.

## Methods

**Fig. 1:**
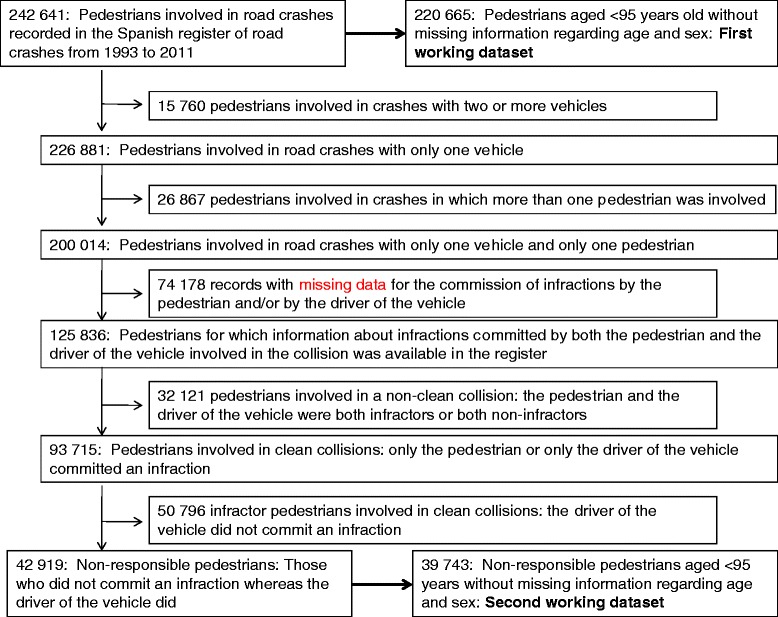
Flowchart illustrating the selection of the subpopulations of pedestrians used in the study

The main source of information for this ecological study was the Spanish Register of Road Crashes with Victims, held by the Spanish General Traffic Directorate. For each traffic crash resulting in injury or death, this police-based register contains information about the nature of the collision and about the vehicles and persons involved. This information is taken from the statistical report and checklist filed for each accident, an official document that the Spanish Traffic Police must complete at the scene of all accidents with victims (Lardelli-Claret et al. 2003). From this database we collected information for all 220 665 pedestrians involved in road crashes in Spain from 1993 to 2011 for which information about their age (up to 94 years old) and gender was recorded. Two of the variables in the database were whether the pedestrian and/or the driver or drivers of the vehicles involved in the collision had committed an infraction (see Additional file 1: Table S1 for the list of infractions recorded for pedestrians and drivers). From this information, we selected a subset of 39 743 pedestrians involved in so-called clean collisions, i.e., those which involved a non-infractor pedestrian and a vehicle whose driver had committed a driving infraction. Figure 1 shows the flowchart of the procedure we used to select this subsample of pedestrians. In accordance with the quasi-induced exposure method (Lenguerrand et al. 2008), we assumed that the age and gender distribution of non-responsible pedestrians involved in clean collisions would approximately resemble that of the overall population of pedestrians exposed to the risk of being struck by a vehicle. For all pedestrians involved in a crash we collected information about their age (<14, 15–24, 25–34, 35–44, 45–54, 55–64, 65–74, 75–84 and 85–94 years), gender and outcome within the first 24 h after the crash: death, severe injury (any person injured in a traffic accident and whose condition required hospitalization for more than 24 h), minor injury (any person injured in a traffic accident and whose condition did not require hospitalization for more than 24 h), no injury (BOE 1993).

The second data source was the Spanish National Institute of Statistics, from which we obtained estimates of the population aged up to 94 years old and stratified according to the same age and gender categories as we used for pedestrians, for each year between 1993 and 2011.

### Analysis

Imagine a subgroup of people of type *i*, defined by their gender and age range. According to decomposition analysis (Dellinger et al. 2002; Li and Baker 1996; Zhu et al. 2013), the PCDR for this subgroup (PCDR_i_) can be obtained by multiplying together three separate rates: the exposure rate (ER_i_: amount of distance or time exposed to the risk of being struck by a vehicle/population), the crash rate (CR_i_: number of collisions/amount of exposure), and the fatality rate (FR_i_: number of deaths after the collisions/number of collisions). If we compare the rates for type *i* people with the corresponding rates for a reference category of people (namely *j*), the model can be expressed as follows:

1 $$ \begin{array}{l}\frac{\mathrm{PCD}{\mathrm{R}}_{\mathrm{i}}}{\mathrm{PCD}{\mathrm{R}}_{\mathrm{j}}}=\frac{\mathrm{E}{\mathrm{R}}_{\mathrm{i}}}{\mathrm{E}{\mathrm{R}}_{\mathrm{j}}}\times \frac{\mathrm{C}{\mathrm{R}}_{\mathrm{i}}}{\mathrm{C}{\mathrm{R}}_{\mathrm{j}}}\times \frac{\mathrm{F}{\mathrm{R}}_{\mathrm{i}}}{\mathrm{F}{\mathrm{R}}_{\mathrm{j}}}\\ {}\kern1em (A)\kern2em (B)\kern1.12em (C)\kern1.24em (D)\end{array} $$

Hereafter we will use term (A) in Eq. (1) to designate the mortality rate ratio of type *i* people (MRR_i_), term (B) to designate the exposure rate ratio of type *i* people (ERR_i_); term (C) to designate the crash rate ratio of people in category *i* (CRR_i_), and term (D) to designate the fatality rate ratio of people in category *i* (FRR_i_). Therefore, Eq. (1) may be rewritten as follows:

2 $$ \mathrm{M}\mathrm{R}{\mathrm{R}}_{\mathrm{i}}=\mathrm{E}\mathrm{R}{\mathrm{R}}_{\mathrm{i}}\times \mathrm{C}\mathrm{R}{\mathrm{R}}_{\mathrm{i}}\times \mathrm{F}\mathrm{R}{\mathrm{R}}_{\mathrm{i}} $$

From the two data sources we used, we can directly obtain two components of Eq. (2), MRRi and FRRi, as follows:

$$ \mathrm{MRRi}=\frac{\frac{\mathrm{Number}\;\mathrm{of}\;\mathrm{deaths}\;\mathrm{of}\;\mathrm{pedestrians}\;\mathrm{in}\;\mathrm{category}\;i}{\mathrm{Total}\;\mathrm{population}\;\mathrm{in}\;\mathrm{category}\;i}}{\frac{\mathrm{Number}\;\mathrm{of}\;\mathrm{deaths}\;\mathrm{of}\;\mathrm{pedestrians}\;\mathrm{in}\;\mathrm{category}\;j}{\mathrm{Total}\;\mathrm{population}\;\mathrm{in}\;\mathrm{category}\;j}} $$

$$ \mathrm{F}\mathrm{R}{\mathrm{R}}_{\mathrm{i}}=\frac{\frac{\mathrm{Number}\;\mathrm{of}\;\mathrm{deaths}\;\mathrm{of}\;\mathrm{pedestrians}\;\mathrm{in}\;\mathrm{category}\;i}{\mathrm{Pedestrians}\;\mathrm{in}\;\mathrm{category}\;i\;\mathrm{in}\mathrm{volved}\;\mathrm{in}\;\mathrm{crashes}}}{\frac{\mathrm{Number}\;\mathrm{of}\;\mathrm{deaths}\;\mathrm{of}\;\mathrm{pedestrians}\;\mathrm{in}\;\mathrm{category}\;j}{\mathrm{Pedestrians}\ \mathrm{in}\ \mathrm{category}\;j\;\mathrm{in}\mathrm{volved}\;\mathrm{in}\ \mathrm{crashes}}} $$

To determine ERRi, the expression that in principal should be used is:

3 $$ \mathrm{E}\mathrm{R}{\mathrm{R}}_{\mathrm{i}}=\frac{\frac{\mathrm{Amount}\kern0.28em \mathrm{of}\kern0.28em \mathrm{pedestrian}\kern0.28em \mathrm{exposure}\kern0.28em \mathrm{of}\kern0.28em \mathrm{people}\kern0.28em \mathrm{in}\kern0.28em \mathrm{category}\kern0.28em i}{\mathrm{Total}\kern0.28em \mathrm{population}\kern0.28em \mathrm{in}\kern0.28em \mathrm{category}\kern0.28em i}}{\frac{\mathrm{Amount}\kern0.28em \mathrm{of}\kern0.28em \mathrm{pedestrian}\kern0.28em \mathrm{exposure}\kern0.28em \mathrm{on}\kern0.28em \mathrm{people}\kern0.28em \mathrm{in}\kern0.28em \mathrm{category}\kern0.28em j}{\mathrm{Total}\kern0.28em \mathrm{population}\kern0.28em \mathrm{in}\kern0.28em \mathrm{category}\kern0.28em j}} $$

However, if the assumption stated above for the subsample of non-infractor pedestrians involved in clean collisions used in our study is correct, the numerator in expression (3) can be estimated as the following quotient:

$$ \mathrm{E}\mathrm{R}{\mathrm{R}}_{\mathrm{i}}=\frac{\frac{\mathrm{Number}\;\mathrm{o}\mathrm{f}\;\mathrm{n}\mathrm{o}\mathrm{n}\hbox{-} \mathrm{infractor}\;\mathrm{pedestrians}\;\mathrm{in}\;\mathrm{category}\;i\;\mathrm{in}\mathrm{volved}\;\mathrm{in}\;\mathrm{clean}\;\mathrm{collisions}}{\mathrm{Total}\;\mathrm{population}\;\mathrm{in}\;\mathrm{category}\;i}}{\frac{\mathrm{Number}\;\mathrm{o}\mathrm{f}\;\mathrm{n}\mathrm{o}\mathrm{n}\hbox{-} \mathrm{infractor}\;\mathrm{pedestrians}\;\mathrm{in}\;\mathrm{category}\;j\;\mathrm{in}\mathrm{volved}\;\mathrm{in}\;\mathrm{clean}\;\mathrm{collisions}}{\mathrm{Total}\;\mathrm{population}\;\mathrm{in}\;\mathrm{category}\;j}} $$

In the above expression, the ratio between the two rates is an unbiased estimate of the relative increase in the exposure rate of people in category *i* relative to people in category *j*.

To obtain CRR_i_, we first calculated, for each group *i*, the unadjusted crash rate (UCR) defined as follows:

$$ \mathrm{U}\mathrm{C}{\mathrm{R}}_{\mathrm{i}}=\frac{\mathrm{Pedestrians}\;\mathrm{in}\;\mathrm{category}\;i\;\mathrm{in}\mathrm{volved}\;\mathrm{in}\;\mathrm{crashes}}{\mathrm{Total}\;\mathrm{population}\;\mathrm{in}\;\mathrm{category}\;i} $$

With the decomposition method again, the following expression can be deduced:

4 $$ \mathrm{U}\mathrm{C}{\mathrm{R}}_{\mathrm{i}}=\mathrm{E}{\mathrm{R}}_{\mathrm{i}}\times \mathrm{C}{\mathrm{R}}_{\mathrm{i}} $$

Dividing both terms in Eq. (4) by the corresponding values for the reference group *j* yields:

5 $$ \frac{\mathrm{UC}{\mathrm{R}}_{\mathrm{i}}}{\mathrm{UC}{\mathrm{R}}_{\mathrm{j}}}=\frac{\mathrm{E}{\mathrm{R}}_{\mathrm{i}}}{\mathrm{E}{\mathrm{R}}_{\mathrm{j}}}\times \frac{\mathrm{C}{\mathrm{R}}_{\mathrm{i}}}{\mathrm{C}{\mathrm{R}}_{\mathrm{j}}} $$

The quotient of UCR_i_/UCR_j_ is the unadjusted crash rate ratio for group *i* (UCRR_i_). Therefore, Eq. (5) can be rewritten as follows:

6 $$ \mathrm{U}\mathrm{C}\mathrm{R}{\mathrm{R}}_{\mathrm{i}}=\mathrm{E}\mathrm{R}{\mathrm{R}}_{\mathrm{i}}\times \mathrm{C}\mathrm{R}{\mathrm{R}}_{\mathrm{i}},\;\mathrm{hence}\;\mathrm{C}\mathrm{R}{\mathrm{R}}_{\mathrm{i}}=\mathrm{U}\mathrm{C}\mathrm{R}{\mathrm{R}}_{\mathrm{i}}/\mathrm{E}\mathrm{R}{\mathrm{R}}_{\mathrm{i}} $$

For the present analysis we selected females <14 years old as the reference category. Poisson regression was used to obtain point estimates, and the corresponding 95 % confidence intervals (95 % CI) of MRR_i,_ ERR_i_, UCRR_i_ and FRR_i_. CRR_i_ were obtained with Eq. (6). To obtain the 95 % CI for this last parameter, a bootstrap procedure was used with 1500 replications. To quantify the association of male gender with MRR, ERR, CRR and FRR within each age group, the corresponding male/female rate ratios were obtained. Finally, to derive the proportion of MRR_i_ attributable to each of the three components (ERR_i_, CRR_i_ and FRR_i_) in each group *i* of people, we first converted the components to their natural logarithms. Then we used the following expressions for each component:

$$ \begin{array}{l}\%{\mathrm{E}}_{\mathrm{i}}:\left[1n\left(\mathrm{E}\mathrm{R}{\mathrm{R}}_{\mathrm{i}}\right)/\left(\left|1n\left(\mathrm{E}\mathrm{R}{\mathrm{R}}_{\mathrm{i}}\right)\right|+\left|1n\left(\mathrm{C}\mathrm{R}{\mathrm{R}}_{\mathrm{i}}\right)\right|+\Big|1n\left(\mathrm{F}\mathrm{R}{\mathrm{R}}_{\mathrm{i}}\right)\right)\right]\times 100\\ {}\%{\mathrm{C}}_{\mathrm{i}}:\left[1n\left(\mathrm{C}\mathrm{R}{\mathrm{R}}_{\mathrm{i}}\right)/\left(\left|1n\left(\mathrm{E}\mathrm{R}{\mathrm{R}}_{\mathrm{i}}\right)\right|+\left|1n\left(\mathrm{C}\mathrm{R}{\mathrm{R}}_{\mathrm{i}}\right)\right|+\Big|1n\left(\mathrm{F}\mathrm{R}{\mathrm{R}}_{\mathrm{i}}\right)\right)\right]\times 100\\ {}\%{\mathrm{F}}_{\mathrm{i}}:\left[1n\left(\mathrm{F}\mathrm{R}{\mathrm{R}}_{\mathrm{i}}\right)/\left(\left|1n\left(\mathrm{E}\mathrm{R}{\mathrm{R}}_{\mathrm{i}}\right)\right|+\left|1n\left(\mathrm{C}\mathrm{R}{\mathrm{R}}_{\mathrm{i}}\right)\right|+\Big|1n\left(\mathrm{F}\mathrm{R}{\mathrm{R}}_{\mathrm{i}}\right)\right)\right]\times 100\end{array} $$

%E_i_, %C_i_ and %F_i_ are, respectively, the percentages of MRR_i_ of each group *i* of people attributable to: i) their exposure as pedestrians, ii) their risk of collision with a vehicle adjusted by their exposure, and iii) their risk of death within the first 24 h after the collision. Unlike previous decomposition studies (Dellinger et al. 2002; Zhu et al. 2013), we only used the absolute values of the natural logarithm of rate ratios in the denominator. Because rate ratios may be higher or lower than 1 depending on the direction of the association of age and gender with each component, our approach yielded negative percentages for some components, whereas the sum of their absolute values was always 100 %. We believe that this procedure better reflects the magnitude and especially the direction in which each component affected overall MRR expressed as a percentage (i.e., in the same direction [positive sign], or in the opposite direction [negative sign] to that of the MRR). All analyses were done with version 12.0 of the Stata statistical package (Stata Corporation 2011).

## Results

Table 1 shows the distribution of pedestrians involved in road crashes and deaths according to age and gender. Table 2 shows the different age and gender distribution of pedestrians depending on their own role (at fault or not at fault) and the role of the driver in each subgroup of collisions. Younger ages and males are overrepresented in the two pedestrian at-fault subgroups. In accordance with the quasi-induced exposure method, the last subgroup of pedestrians (non-infractor pedestrians and infractor drivers) was used to obtain the ERR. In this subgroup, the proportion of females and older pedestrians was higher than in the other three subgroups.

**Table 1:** Distribution of pedestrians involved in road crashes and deaths according to age and gender. Spain, 1993–2011

	Males		Females	
Age (years)	All pedestrians involved in crashes	Death	All pedestrians involved in crashes	Deaths
0–14	20 810	310	13 765	191
15–24	14 237	532	14 318	236
25–34	14 415	859	11 475	242
35–44	12 471	920	10 230	224
45–54	11 239	954	11 334	276
55–64	11 549	972	12 901	420
65–74	13 411	1145	15 909	817
75–84	11 525	1187	14 084	943
85–94	3408	388	3584	323
Total	113 065	7267	107 600	3672

**Table 2:** Distribution of pedestrians by age and gender group according to the role of the pedestrian and the driver involved in the collision

	Driver not at fault/Pedestrian not at fault				Driver not at fault/Pedestrian at fault				Driver at fault/Pedestrian at fault				Driver at fault/Pedestrian not at fault^a^			
	Males		Females		Males		Females		Males		Females		Males		Females	
Age (years)	*N*	%	*N*	%	*N*	%	*N*	%	*N*	%	*N*	%	*N*	%	*N*	%
0–14	925	18.2	559	11.2	7323	25.8	3847	20.4	1644	16.5	1016	11.2	2106	12.0	1744	7.9
15–24	600	11.8	586	11.7	3316	11.7	2562	13.6	1092	10.9	977	10.8	1703	9.7	2550	11.5
25–34	715	14.1	575	11.5	3223	11.3	1707	9.0	1146	11.5	687	7.6	2056	11.7	2250	10.2
35–44	604	11.9	535	10.7	3022	10.6	1425	7.5	978	9.8	710	7.9	1791	10.2	2134	9.6
45–54	523	10.3	583	11.7	2625	9.2	1619	8.6	1021	10.2	913	10.1	1869	10.6	2624	11.9
55–65	530	10.4	635	12.7	2654	9.3	2049	10.8	1109	11.1	1127	12.5	2112	12.0	2987	13.5
65–74	499	9.8	681	13.6	3031	10.7	2776	14.7	1294	13.0	1635	18.1	2793	15.9	3597	16.2
75–84	508	10.0	632	12.6	2458	8.7	2379	12.6	1286	12.9	1577	17.4	2473	14.0	3368	15.2
>84	173	3.4	213	4.3	756	2.7	534	2.8	415	4.2	402	4.4	703	4.0	883	4.0
Total	5077	100.0	4999	100.0	28 408	100.0	18 898	100.0	9985	100.0	9044	100.0	17 606	100.0	22 137	100.0

^a^This last group of pedestrians was used to obtain exposure rate ratios

**Fig. 2:**
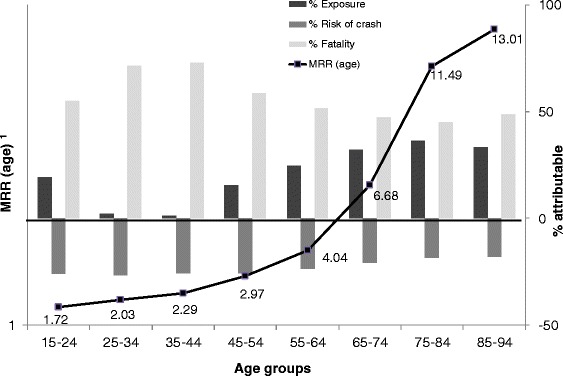
Decomposition of mortality rate ratio (MRR) for each age group into the proportion attributable to each component. ^1^ Reference: 0–14 years old

**Fig. 3:**
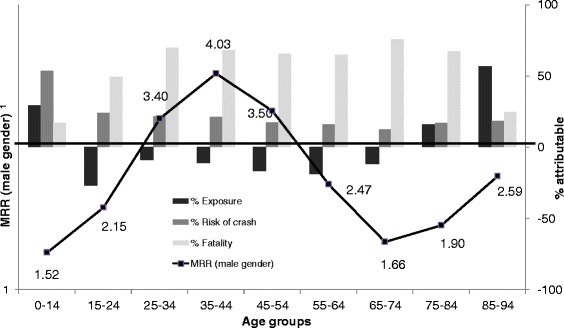
Decomposition of mortality rate ratio (MRR) for male gender in each age group into the proportion attributable to each component. ^1^ Reference: Females

Table 3 shows the MRR for each age and gender category for the whole study period. As expected, MRR increased sharply with age in the whole population (Fig. 2) and also in each gender separately. Furthermore, MRR for each age group was always higher for males, with male/female rate ratios (Fig. 3) increasing from 1.52 in the lowest age category to 4.03 in the 35–44 year age group, then decreasing to 1.66 in the 65–74 year age group, and finally increasing again to 2.59 in the oldest group.

**Table 3:** Mortality rate ratios (MRR) for each age and gender group

	Both genders			Females			Males			MRR males/MRR females
Age (years)	MRR	95 % CI		MRR	95 % CI		MRR	95 % CI		
0–14	1.00	Reference		1.00	Reference		1.52	1.27	1.82	1.52
15–24	1.72	1.54	1.93	1.37	1.14	1.66	2.95	2.50	3.49	2.15
25–34	2.03	1.82	2.25	1.16	0.96	1.40	3.93	3.36	4.60	3.40
35–44	2.29	2.06	2.54	1.15	0.95	1.39	4.62	3.95	5.40	4.03
45–54	2.97	2.68	3.30	1.68	1.40	2.02	5.90	5.05	6.89	3.50
55–64	4.04	3.65	4.48	3.00	2.53	3.56	7.41	6.34	8.65	2.47
65–74	6.68	6.05	7.36	6.50	5.55	7.61	10.81	9.28	12.60	1.66
75–84	11.49	10.43	12.67	10.73	9.19	12.54	20.40	17.51	23.77	1.90
85–94	13.01	11.60	14.58	10.97	9.17	13.12	28.46	23.93	33.84	2.59

Table 4 shows the results of the decomposition analysis for MRR in each age and gender category. When both genders were considered together, increasing age was strongly related with higher values for FRR and, to a lesser extent (from 45 to 54 years old onward), with higher values for ERR. The opposite trend was observed for CRR. The same pattern of associations was observed within each gender. When the values for males were divided by the corresponding values for females in each age stratum, higher rate ratios were found in males for CRR and especially for FRR and in all groups between the ages of 15 and 64 years. However, the values for ERR were higher for women in all age groups except at extreme ages (0–14 and >74 years old).

**Table 4:** Decomposition of mortality rate ratios (MRR) for each age and gender group

Decomposition of MRR for age groups											Decomposition of MRR for male gender	
3.1. Exposure rate ratios											ERR males/ERR females	
	Both genders				Females			Males				
Age (years)	ERR^a^	95 % CI		%E^b^	ERR	95 % CI		ERR	95 % CI			%E
0–14	1	Reference			1	Reference		1.13	1.06	1.21	1.13	29.48
15–24	1.24	1.19	1.29	19.11	1.63	1.53	1.73	1.04	0.97	1.11	0.64	−27.03
25–34	1.03	0.99	1.08	2.06	1.18	1.11	1.25	1.03	0.97	1.1	0.88	−8.94
35–44	1.02	0.98	1.07	1.18	1.2	1.12	1.27	0.99	0.92	1.05	0.82	−10.91
45–54	1.41	1.35	1.48	15.39	1.75	1.65	1.86	1.27	1.19	1.35	0.72	−17.08
55–64	1.93	1.85	2.01	24.74	2.33	2.2	2.48	1.76	1.65	1.88	0.75	−19.17
65–74	2.83	2.72	2.95	32.09	3.13	2.96	3.32	2.89	2.72	3.07	0.92	−12.10
75–84	4.1	3.94	4.27	36.41	4.2	3.96	4.45	4.65	4.38	4.95	1.11	16.07
85–94	3.78	3.56	4.00	33.31	3.28	3.03	3.56	5.65	5.17	6.16	1.72	56.84
3.2. Risk of crash rate ratios											CRR males/CRR females	
	Both genders				Females			Males				
Age (years)	CRR^c^	95 % CI		%C^d^	CRR	95 % CI		CRR	95 % CI			%C
0–14	1	Reference			1	Reference		1.25	1.17	1.34	1.25	53.59
15–24	0.75	0.71	0.78	−25.87	0.71	0.66	0.76	1.06	0.98	1.14	1.49	23.87
25–34	0.67	0.64	0.7	−26.59	0.65	0.6	0.69	0.89	0.83	0.95	1.37	21.36
35–44	0.64	0.61	0.68	−25.78	0.61	0.56	0.65	0.88	0.82	0.95	1.45	20.94
45–54	0.56	0.54	0.59	−25.79	0.55	0.51	0.58	0.76	0.71	0.82	1.39	17.37
55–64	0.53	0.51	0.56	−23.66	0.55	0.51	0.58	0.69	0.64	0.74	1.27	16.08
65–74	0.51	0.49	0.53	−20.72	0.56	0.53	0.6	0.61	0.57	0.65	1.09	12.23
75–84	0.49	0.47	0.51	−18.50	0.53	0.5	0.56	0.59	0.55	0.63	1.11	16.87
85–94	0.49	0.46	0.52	−17.84	0.51	0.47	0.56	0.61	0.56	0.68	1.19	18.64
3.3. Fatality rate ratios											FRR males/FRR females	
	Both genders				Females			Males				
Age (years)	FRR^e^	95 % CI		%F^f^	FRR	95 % CI		FRR	95 % CI			%F
0–14	1	Reference			1	Reference		1.07	0.9	1.29	1.07	16.93
15–24	1.86	1.66	2.08	55.02	1.19	0.98	1.44	2.69	2.28	3.18	2.27	49.09
25–34	2.93	2.64	3.26	71.35	1.52	1.26	1.84	4.29	3.67	5.02	2.83	69.70
35–44	3.48	3.13	3.86	73.03	1.58	1.3	1.91	5.32	4.55	6.21	3.37	68.15
45–54	3.76	3.39	4.17	58.82	1.75	1.46	2.11	6.12	5.24	7.15	3.49	65.55
55–64	3.93	3.55	4.35	51.60	2.35	1.98	2.78	6.07	5.19	7.08	2.59	64.75
65–74	4.62	4.19	5.09	47.20	3.7	3.16	4.33	6.15	5.28	7.17	1.66	75.67
75–84	5.74	5.21	6.33	45.09	4.83	4.13	5.64	7.42	6.37	8.65	1.54	67.06
85–94	7.02	6.26	7.87	48.85	6.49	5.43	7.77	8.2	6.9	9.76	1.26	24.52

^a^ *ERR* exposure rate ratio

^b^ *%E* proportion of MRR attributable to ERR

^c^ *CRR* crash rate ratio

^d^ *%C* proportion of MRR attributable to CRR

^e^ *FRR* fatality rate ratio

^f^ *%F* proportion of MRR attributable to FRR

Table 4 and Figs. 2 and 3 also show the relative percent contribution of each of the three components of MRR obtained for each age and gender group. The main determinant of the increases in MRR associated with age was FRR (Fig. 2), although its relative contribution decreased with age from more than 70 % in the 25–44 year age groups to less than 50 % from 65 years on. The relative contribution of ERR was lower, increasing with age up to more than 30 % in the oldest age groups. Finally, because CRR values tended to decrease with age, their relative contributions to MRR values were negative, decreasing slightly with age. Regarding the excess MRR for male gender (Fig. 3), its main determinant in all age strata was again FRR except in the extreme age groups, with percentages higher than 65 % for age groups between 25 and 84 years. CRR also contributed to this male excess in MRR, but to a smaller degree (less than 25 %) except in the 0–14 year age group, in which CRR contributed 54 % of the excess MRR for male gender. The contribution of ERR was small and negative in all but extreme ages: in the oldest age group, ERR made a large positive contribution (57 %) to the excess MRR for male gender.

## Discussion

Our results reveal a strong association between PCDR and both increased age and male gender, in accordance with almost all previous studies (Chang 2008; Mabunda et al. 2008). The association between death and age is strongly related with increased fatality in older pedestrians, in agreement with many previous studies (Chang 2008; Henary et al. 2006; Kim et al. 2008; Mohamed et al. 2013; Tefft 2013); this association is usually explained by the increased frailty associated with ageing (Kim et al. 2008; Tarko and Azam 2011). However, the role of the other two components (i.e., exposure and risk of crash) remains uncertain, partially because it is difficult to determine the amount of exposure in pedestrians accurately (Clifton and Livi 2005; Keall 1995). Unlike previous studies (Keall 1995; Milligan et al. 2013; Mindell et al. 2012; Zhu et al. 2013), we used a quasi-induced exposure method based on comparisons of the age and gender distribution of “innocent” pedestrians involved in clean collisions with age and gender in the whole population. In doing so, we took into consideration only the exposure windows in which pedestrians can be considered at fault or not at fault for a collision (i.e., while they are crossing or walking along a roadway), and therefore at risk of being struck by a vehicle. With this method, the amount of exposure increased with age from 45 years old onward; therefore, it also contributed to the direct association between ageing and higher PCDR. This result is not surprising: older pedestrians are exposed to the risk of collision with a vehicle for longer periods because they need more time to walk the same length-at-risk (typically, crossing a road) (Avineri et al. 2012; Hoxie and Rubenstein 1994; Keall 1995; Oxley et al. 1997; Romero-Ortuno et al. 2010). These results thus emphasize the need to develop interventions intended to compensate for this handicap in older pedestrians, by (for example) lengthening traffic light times for crossing (Hoxie and Rubenstein 1994; Romero-Ortuno et al. 2010) or building median strips to facilitate crossing two-lane roads (Oxley et al. 1997).

Another interesting finding from our study is the inverse association between age and the risk of involvement in a crash adjusted by exposure. As widely reported for pedestrians and other types of road users (Cestac et al. 2011; Ivers et al. 2009; Sullman et al. 2011), this risk is higher for the youngest pedestrians. Pollack et al. reported that younger pedestrians, such as students, engage in risky behaviors in terms of distracted walking (e.g., walking while talking or texting on a cell phone, or listening to music on an iPod) (Pollack et al. 2014). Furthermore, in our study the lowest risk is seen in the oldest pedestrians. This is in agreement with previous findings that older people take fewer risks (Bernhoft and Carstensen 2008), are less likely to attempt to cross in risky situations (Holland and Hill 2007), and adopt safer behaviors when crossing a street (Keall 1995; Oxley et al. 1997).

Regarding the role of gender, our results are also in agreement with previous studies that found higher PCDR in males than females (Chang 2008; Zhu et al. 2013), although the strength of this association changed with age. Decomposition analysis revealed, in accordance with a study by Zhu et al. (2013), that fatality is again the most important determinant of the higher PCDR in males. This finding has also been observed in previous studies (Chang 2008; Clifton et al. 2005; Zhu et al. 2013), although the reasons for this association are not well understood. One hypothesis is that compared to females, male pedestrians are involved in collisions of higher intrinsic severity. For example, it has been reported that collisions at night (in which males may be involved more frequently because females tenfd to walk less at night than males (Clifton and Livi 2005) are more severe than those that occur during daylight (Kim et al. 2008).

In accordance with previous studies (Keall 1995; Zhu et al. 2013), we also found that the risk of involvement in a crash adjusted by exposure is higher in male pedestrians than in females, especially in younger age groups. It has been shown that pedestrian women are more sensitive to traffic safety than men, and appear to engage in fewer risk-taking behaviors (Clifton et al. 2005; Clifton and Livi 2005; Holland and Hill 2007; Sullman et al. 2011). Ulfarsson et al. observed that male pedestrians were more likely to be solely at fault than females in pedestrian–motor vehicle crashes (Ulfarsson et al. 2010). Furthermore, Faria et al. (2010) found that males tended to follow others pedestrians crossing a road more frequently than females. The higher fatality and risk of involvement in a crash in males compared to females is partially counterbalanced by their lower amount of exposure, observed for males of all ages except the extreme age groups. This may be related with both the amount of walking, which is greater in females according to some studies (Keall 1995), and with the speed of walking, which is faster in males than in females (Avineri et al. 2012; Miguel 2013).

Our study has several limitations. First, we used a police-based registry. Hypothetically, and as noted in other countries (Lopez et al. 2000; Sciortino et al. 2005), the Spanish registry tends to underestimate less severe crashes and collisions in urban areas. This is especially relevant in collisions involving pedestrians, because they occur mainly in urban areas. We must therefore assume that we used a biased sample of crashes involving pedestrians: the pattern depicted by our decomposition model may be more applicable to severe crashes. However, severe crashes are those of greatest concern from a public health perspective, because they should constitute the primary focus of preventive interventions. The overrepresentation of more severe crashes (i.e., those that resulted in deaths or severe injuries) in police databases may also lead to frailty bias (Langford et al. 2006), which would lead to overestimation of the ERR for the oldest pedestrians, and underestimation of their FRR.

Furthermore, as shown in the flowchart in Fig. 1, we were obliged to exclude a number of pedestrians in the original database because the traffic police had not recorded whether they and/or the driver involved in the crash had committed an infraction. Most of these missing cases occurred in specific provinces (and from specific years onward) where the traffic police were not required to record this information. We can offer no hypotheses regarding the direction or magnitude of this possible selection bias. Moreover, the Spanish register includes only deaths that occur within the first 24 h after a crash. The subsequent underestimation of fatality rates may differ for different age groups if we assume that increasing age is related with a worse prognosis for crash-related injuries.

Several concerns have been raised about quasi-induced exposure methods when applied to other road users (Jiang and Lyles 2010). For example, it may be difficult to identify the party responsible for the crash from information in the police-based registry regarding whether an infraction was committed, and whether the infractor was the pedestrian or the driver of the vehicle. It is important to emphasize that the way we assigned responsibility was not deterministic, but probabilistic: in a collision between a pedestrian who had not committed an infraction and a vehicle whose driver had just committed an infraction, the driver was much more likely to be at fault for the collision than the pedestrian. Doubts may also arise regarding the validity of our assumption that the age and gender distribution in our sample of non-responsible pedestrians is representative of the whole population of exposed pedestrians. In this connection it is important to emphasize that our estimate of exposure did not include the whole time or the entire distance walked on sidewalks or pedestrian streets. Finally, the decomposition method assumes that each conditional probability is independent of and not confounded with other probabilities, whereas in fact the effects are likely to overlap (Goldstein et al. 2011) and depend upon the classified groups being relatively homogenous.

## Conclusion

In conclusions, our decomposition procedure allowed us to disentangle the association of pedestrian’s age and gender with their PCDR. This is important since the magnitude and direction of the associations were different (and in some cases, opposite) depending on the specific component of PCDR being explored. From a practical viewpoint our results may help to identify priority areas for both public health and research. According to their mortality rates, it seems clear that males, and especially older pedestrians, are priority groups for whom preventive measures should be targeted. Furthermore, the emphasis should be on decreasing pedestrian fatality rates, because these rates are the main factor responsible for age and gender differences in mortality rates. Therefore, interventions focused on decreasing the intrinsic severity of crashes (e.g., reducing the speed limit for vehicles in urban areas, conspicuous stop signs or improved visibility at intersections) (DiMaggio et al. 2014) should be a priority. On the other hand, the high fatality rate in older pedestrians may be difficult to decrease, because it is probably related to their increased frailty. Therefore, this subgroup of pedestrians may benefit from strategies focused on their exposure, which also plays an important role in their high mortality rates. Interventions for this group should therefore focus on providing enough time for them to cross roads safely (e.g., longer crossing times for pedestrians at traffic lights, so that pedestrians can cross safely before traffic begins to move again) (Redmon 2011). Finally, the high risk of collision in the youngest pedestrians (i.e., from 0 to 14 years old) should be taken into consideration in order to reinforce educational interventions (e.g., a communications campaign on pedestrian risks to improve awareness and knowledge about how to travel safely as a pedestrian) (Violano et al. 2015) and legislative interventions targeted at this population group (e.g., road safety education as a mandatory and examinable subject in schools, including sessions on pedestrian safety) (Redmon 2011; Pollack et al. 2014).

## Additional file

Additional file 1: Table S1. List of infractions recorded by the Spanish General Traffic Directorate. (DOCX 17 kb)

## Abbreviations

CR: crash rate
CRR: crash rate ratio
ER: exposure rate
ERR: exposure rate ratio
FR: fatality rate
FRR: fatality rate ratio
MRR: mortality rate ratio
PCDR: pedestrian crash death rate
UCR: Unadjusted crash rate
UCRR: unadjusted crash rate ratio
