# Erratum to: Gender and age differences in components of traffic-related pedestrian death rates: exposure, risk of crash and fatality rate

**DOI:** 10.1186/s40621-016-0085-4

**Published:** 2016-08-09

**Authors:** María Ángeles Onieva-García, Virginia Martínez-Ruiz, Pablo Lardelli-Claret, José Juan Jiménez-Moleón, Carmen Amezcua-Prieto, Juan de Dios Luna-del-Castillo, Eladio Jiménez-Mejías

**Affiliations:** 1 Doctorate Program in Clinical Medicine and Public Health, University of Granada, Granada, Spain; 2 Department of Preventive Medicine and Public Health, School of Medicine, University of Granada, Avda. de la Investigación, 11, 18016 Granada, Spain; 3 Centros de Investigación Biomédica en Red de Epidemiología y Salud Pública (CIBERESP), Barcelona, Spain; 4 Instituto de Investigación Biosanitaria de Granada, C/Doctor Azpitarte 4 4a Planta, Edificio Licinio de la Fuente, 18012 Granada, Spain; 5 Department of Biostatistics, School of Medicine, University of Granada, Avda. de la Investigación, 11, 18016 Granada, Spain

After the publication of this work (Onieva-García et al. 2016) the funder of the project that supports this study requested that in the section ‘Acknowledgements’ we add the institution: Institute of Health Carlos III. The revised text reads as follows:
