# Supplementary material for: Gender and age differences in components of traffic-related pedestrian death rates: exposure, risk of crash and fatality rate
Source: Inj Epidemiol. 2016 Jun 10;3(1):14. doi: 10.1186/s40621-016-0079-2 (PMC4901119; doi:10.1186/s40621-016-0079-2)
Supplement: Additional file 1: Table S1. — List of infractions recorded by the Spanish General Traffic Directorate. (DOCX 17 kb) [file 40621_2016_79_MOESM1_ESM.docx]

**Table S1**. List of infractions recorded by the Spanish General Traffic Directorate

Pedestrian infractions:

|  | n^1^ | %^1^ |
| --- | --- | --- |
| Not respecting a pedestrian crossing sign | 6379 | 9.62 |
| Not using a pedestrian crosswalk | 18860 | 28.43 |
| Disobeying instructions from a traffic police officer | 95 | 0.14 |
| Entering or crossing a roadway in violation of the traffic code | 31452 | 47.41 |
| Occupying or traveling on a roadway in violation of the traffic code | 4102 | 6.18 |
| Occupying or traveling on a sidewalk in violation of the traffic code | 555 | 0.84 |
| Entering or exiting the vehicle in violation of the traffic code | 312 | 0.47 |
| Other infractions | 4580 | 6.9 |

1: Values and percentages are from infractor pedestrians without missing values for age and sex

Driver infractions:

| 1. Speed-related infractions |
| --- |
| Inappropriate speed for existing conditions  Excessive speed  Driving too slowly |
| 2. Other infractions |
| Distracted or inattentive  Incorrect use of vehicle lights  Driving in the wrong lane or in the wrong direction  Partially crossing into the opposite lane  Incorrect turn  Illegal passing  Zig-zagging  Violating the minimum safety distance between vehicles  Unjustified braking  Failure to grant right-of-way  Disobeying a traffic light  Disobeying a stop sign  Disobeying a yield sign  Entering a pedestrian crossing  Disobeying a traffic sign or a police signal  Failure to correctly signal intention  Joining traffic flow carelessly  Stopping where no stopping is allowed or in a dangerous place  Opening vehicle door into traffic  Other infraction |
